# A novel surveillance approach for disaster mental health

**DOI:** 10.1371/journal.pone.0181233

**Published:** 2017-07-19

**Authors:** Oliver Gruebner, Sarah R. Lowe, Martin Sykora, Ketan Shankardass, S. V. Subramanian, Sandro Galea

**Affiliations:** 1 Harvard T.H. Chan School of Public Health, Department of Social and Behavioral Sciences, Boston, MA, United States of America; 2 Montclair State University, Department of Psychology, Montclair, NJ, United States of America; 3 Loughborough University, School of Business and Economics (SBE), Centre for Information Management (CIM), Loughborough, United Kingdom; 4 Wilfrid Laurier University, Department of Health Sciences, Waterloo, Ontario, Canada; 5 Boston University, School of Public Health, Boston, MA, United States of America; New York City Department of Health and Mental Hygiene, UNITED STATES

## Abstract

**Background:**

Disasters have substantial consequences for population mental health. Social media data present an opportunity for mental health surveillance after disasters to help identify areas of mental health needs. We aimed to 1) identify specific basic emotions from Twitter for the greater New York City area during Hurricane Sandy, which made landfall on October 29, 2012, and to 2) detect and map spatial temporal clusters representing excess risk of these emotions.

**Methods:**

We applied an advanced sentiment analysis on 344,957 Twitter tweets in the study area over eleven days, from October 22 to November 1, 2012, to extract basic emotions, a space-time scan statistic (SaTScan) and a geographic information system (QGIS) to detect and map excess risk of these emotions.

**Results:**

Sadness and disgust were among the most prominent emotions identified. Furthermore, we noted 24 spatial clusters of excess risk of basic emotions over time: Four for anger, one for confusion, three for disgust, five for fear, five for sadness, and six for surprise. Of these, anger, confusion, disgust and fear clusters appeared pre disaster, a cluster of surprise was found peri disaster, and a cluster of sadness emerged post disaster.

**Conclusions:**

We proposed a novel syndromic surveillance approach for mental health based on social media data that may support conventional approaches by providing useful additional information in the context of disaster. We showed that excess risk of multiple basic emotions could be mapped in space and time as a step towards anticipating acute stress in the population and identifying community mental health need rapidly and efficiently in the aftermath of disaster. More studies are needed to better control for bias, identify associations with reliable and valid instruments measuring mental health, and to explore computational methods for continued model-fitting, causal relationships, and ongoing evaluation. Our study may be a starting point also for more fully elaborated models that can either prospectively detect mental health risk using real-time social media data or detect excess risk of emotional reactions in areas that lack efficient infrastructure during and after disasters. As such, social media data may be used for mental health surveillance after large scale disasters to help identify areas of mental health needs and to guide us in our knowledge where we may most effectively intervene to reduce the mental health consequences of disasters.

## Introduction

Human-made and natural disasters happen with regularity around the globe and are increasing with the growing influence of environmental climate change [1,2]. Together with the current upsurge of violent conflicts, war, and terrorism, these life-threatening events put an increasing number of communities at risk for experiencing the mental health consequences of disasters, with post traumatic stress disorder (PTSD) and depression being the most commonly reported problems [3–5]. Exposure factors for post disaster mental health include female gender, low socioeconomic status (SES), minority status, lack of social support, and pre disaster mental health problems, along with traumatic experiences (actual or threatened death, serious injury, or sexual violation) and stressors (e.g., lack of food, water, medical care, or displacement) [3,4,6–11].

However, the existing literature on the mental health consequences of disaster suffers from at least three major limitations. First, little is known about the multiple dimensions of negative emotional reactions in the disaster context. For example, anger, disgust, fear, and sadness, according to Ekman [12], have been shown to differ in their antecedent events and likely behavioral responses, can be seen as an adaptive to a person dealing with an immediate danger (e.g., a hurricane), experiencing an irrevocable loss (e.g., personal belongings, job, life), or the expectation of failure to achieve a goal (e.g., as experienced with fear). Furthermore, these basic emotions have been shown to be associated with autonomic nervous system (ANS) responses [12]. Most studies in the disaster context however have combined these negative emotional reactions in an affirmative index with higher values being consistently associated with negative consequences of disaster (e.g., PTSD and depression) [13,14]. Therefore, little is known about the differential distributions of multiple dimensions of emotions in the context of disaster. Assessing multiple dimensions of emotions may hence inform about *how* a disaster is experienced in affected populations.

Second, research to date has provided limited evidence on the temporal dimensions of negative emotions in the context of disaster. For example, we do not know much about emotional reactions in the advent or during a disaster, as surveys often collect information post disaster, thereby missing important pre or peri event information. Research has shown that peri event traumatic experience (e.g., multiple losses) is highly interrelated with acute stress (e.g., expressed through intense fear) during and in the immediate aftermath of a disaster, which in turn has been shown to be predictive of posttraumatic stress disorder (PTSD) [3,4,15]. This may also be relevant in the advent of a prospective disaster, such as a hurricane (which may be known by an estimated track forecast), that may increase fear in affected populations. Furthermore, acute stress may impair social, occupational, or other important areas of pre disaster functioning, which has also been shown to predict post disaster mental health [3]. Hence, assessing the distribution of emotional reactions pre, peri, and post disaster may inform about the temporal dimensions of distress, that is, the times *when* a disaster is considered most severe in affected populations.

Third, there is a paucity of research that has investigated the geographic concentrations of negative emotions pre, peri, or post disaster. Studies of this kind may reveal areas and times of excess risk of negative emotional reactions, that is, information about *where* and *when* the population exhibits such responses in the context of disaster.

Information from social media has potential to complement traditional survey techniques as it provides fine-grained measurements of behavior over time, while taking advantage of large population sample sizes (e.g., the user community of Twitter, Facebook, or YouTube). For example, social media data has been increasingly used for understanding population health. Paul and Dredze [16,17] developed a topic model for Twitter to discover associated symptoms, treatments, and general words with diseases. Althouse et al. [18] investigated population health concerns during the United States' Great Recession and Yang et al. [19] used Twitter to track the diffusion of information with regard to disease outbreaks in and across different cities and geographic regions. Furthermore, mental health has been investigated in Twitter and screening instruments for population mood or emotions have been usefully implemented in several studies [20–36]. Evidence exists that Twitter can be used to identify the sentiment (positive or negative) of Twitter messages based on text classification [26,34]. Thelwall et al. [30] used sentiment analysis to find evidence for an association between discussion of important events in Twitter and average negative sentiment. Bollen et al. [27] modeled public mood and emotion with Twitter sentiment and socioeconomic phenomena and found that social, political, cultural, and economic events were correlated with public mood levels along a range of different mood dimensions. Furthermore, DeChoudhury et al. [31] analyzed changes in Twitter users’ affective reactions to violence during the Mexican drug war 2010–12. In addition, there is recent evidence that rigorous application of even simple Natural Language Processing (NLP) methods to social media data can yield insights into mental health disorders [22,35], such as PTSD [36] and depression [29,32,33]. Initial work from computational linguists and psycholinguistics revealed that some indicators of mental health based on, for example, frequencies of first and third person pronouns, anger words, varied negative emotions, and related patterns of language use, are significantly associated with Twitter users’ likelihood of self-reported mental health problems [36]. However, despite these advances, research to date lacks the detection of specific emotions from social media, especially in the disaster context. Assessing multiple dimensions of emotional reactions may shed light on how disasters are experienced within affected communities.

Assessment of social media is further promising in an area where traditional surveillance is largely incomplete. For example, temporal gaps in regularly conducted surveys exist because such studies are not specific to the health event under investigation, such as a hurricane. Event-specific surveys, in contrast, only cover a small proportion of the population and are normally conducted post-event and, as such, miss important pre-event information. Social media may also provide information where traditional surveys are restricted because of limited financial resources. However, cautionary steps must be taken in the interpretation of social media analysis, as high profile automated surveillance systems such as Google Flu Trends have been found inferior to already strong traditional surveillance [37,38]. Therefore, novel surveillance efforts should be informed by the track record of past systems. For example, data from Twitter have been used to identify populations in need of short-term relief and to guide planning and response before, during, and after crises and disasters [16,26,39–44]. Assessing emotional reactions over time may shed light on when disasters are considered most severe within affected communities.

Furthermore, space-time syndromic surveillance has been shown effective in the detection of disease outbreaks indicating specific areas and populations with excess risk over time [45–48]. For example, Nagar et al. [45] used Twitter data to identify the spatial distribution of primary outbreak clusters of high-probability influenza-like illness emergency department visits. Sakaki et al. [43] analyzed Twitter tweets and showed that disaster detection and the location or path of these events can be estimated based on Twitter users’ activity. The same was true in a study of Kryvasheyeu et al. [44] who found a strong relationship between proximity to Hurricane Sandy’s path and hurricane-related Twitter activity. However, space-time analysis of extracted mental health indicators from social media has only been minimal exploited so far, that is, we do not know much about specific areas and populations within them that have increased risk of mental health indicators in relation to certain events or crises. Scholars have only recently begun to combine social media analysis and space-time surveillance for the early identification of populations at risk for the mental health consequences of traumatic events [49]. Assessing the geographic variability of emotional reactions over time may shed light on where and when disasters are considered most severe within affected communities.

Therefore, we set out to demonstrate the feasibility of a space-time syndromic surveillance approach for mental health using geo-located Twitter data from the greater New York City (NYC) area during Hurricane Sandy, one of the most destructive hurricanes in United States history, which formed over the Caribbean on October 22 and made landfall in the New York City (NYC) area on October 29, 2012 [5]. Specifically, we aimed to 1) extract basic emotions from Twitter for the study area over eleven days, i.e., October 22 to November 1, 2012, and to 2) detect space-time clusters representing excess risk of these emotional reactions as a way anticipating acute stress in the population.

## Methods

Data included geo-located, English language tweets from Twitter within a rectangular grid around NYC within eleven days around the time when Hurricane Sandy made landfall (October 29, 2012), i.e., between October 22 and November 1, 2012. Data was obtained from the Harvard Center for Geographical Analysis Geo-tweet archive (CGA) and combined with additional Twitter data from GeoFeedia (https://geofeedia.com) to fill some missing dates of the CGA data. Although we aimed to compile data covering a 14-day cycle that would have captured the typical timeframe in many standard psychiatric questionnaires, our combined data using these sources restricted us to only 11 days. The combined dataset included 423,931 tweets, of which 344,957 tweets (81%) were deemed suitable for our analysis, i.e., were in English as identified by an ensemble vote of two language identification models [50,51].

We followed the guidelines and recommendations to assure Good Epidemiological Practice (GEP) as defined by the German Society for Epidemiology [52]. The study was therefore conducted in accordance with ethical principles and respected human dignity as well as human rights. All information was stored and used anonymously in our analysis. Furthermore, this study complied with the terms of services of the data providers.

First, we used a semantic modeling approach that has been increasingly applied in sentiment analysis due to the rich expressivity, performing well over purely dictionary-based approaches [34]. Specifically, we developed and applied advanced sentiment detection that is implemented in EMOTIVE and detailed elsewhere [24]. Briefly, EMOTIVE analysis allows for the capture of Ekman’s cross-cultural basic emotions [53] (e.g., anger, disgust, fear) by combining a natural language processing (NLP) pipeline with a detailed semantic model, containing over 1,000 explicit emotional expressions with various associated language elements, including a wide set of intensifiers, conjunctions, negators, interjections and linguistic analysis rules represented in a formal semantic model (i.e. Resource Description Framework [RDF], Resource Description Framework Schema [RDFS], and Web Ontology Language [OWL] ontology). The EMOTIVE ontology has been evaluated with standard measures, such as recall and precision from information retrieval field, and was found to perform well against other systems, and currently holds the highest reported f-measure for fine-grained emotion expression detection [24]. For this study, we first used EMOTIVE to detect seven specific emotions in tweets, such as anger, confusion, disgust, fear, sadness, shame, and surprise. Thereby, for each tweet, EMOTIVE added a new column with the number of times each emotion is identified within the tweet. For each tweet, we then identified whether an emotion was detected and coded dichotomously for the presence (case = 1) or absence (no case = 0) of the basic emotion. A dataset for each emotion was then created only containing the cases representing the basic emotion classified by EMOTIVE (along with the original information from the tweet, such as geo location, time, or tweet text). In addition to the already mentioned evaluation of the accuracy and performance of EMOTIVE, a brief qualitative manual review of a sample of EMOTIVE’s output showed a consistent and correctly categorized set of emotions among the seven basic emotions.

Second, a formal space-time scan statistic was applied to detect clusters of excess risk of the EMOTIVE-classified emotions (case = 1) retrospectively in space and time, with no population at risk information needed using the space-time permutation model [48]. The scan statistic is available in the free software SaTScan^TM^ that detects clusters by gradually moving a scanning window with a circular cylinder across time and space, noting the number of observed and expected observations (emotions) inside the cylinder at each location. A cluster in a particular geographic area is detected if, during one day, that area has a higher proportion of emotions in that time period compared to the remaining geographic areas [54]. Relative risk (RR) for a significant cluster is calculated as the observed number of cases divided by the expected number of cases, with RR > 1 indicating that the observed number of emotions is greater than expected [55]. Statistical inference was evaluated by a Monte Carlo hypothesis test with 999 replications.

Third, significant space-time clusters were mapped and a point density heat map was created for significant cluster locations in order to show the spatial distribution (point density) of tweets within the cluster while preserving anonymity of the point location itself. For mapping, we used the freely available geographic information system QGIS (QGIS Development Team, 2016).

## Results

**Fig 1:**
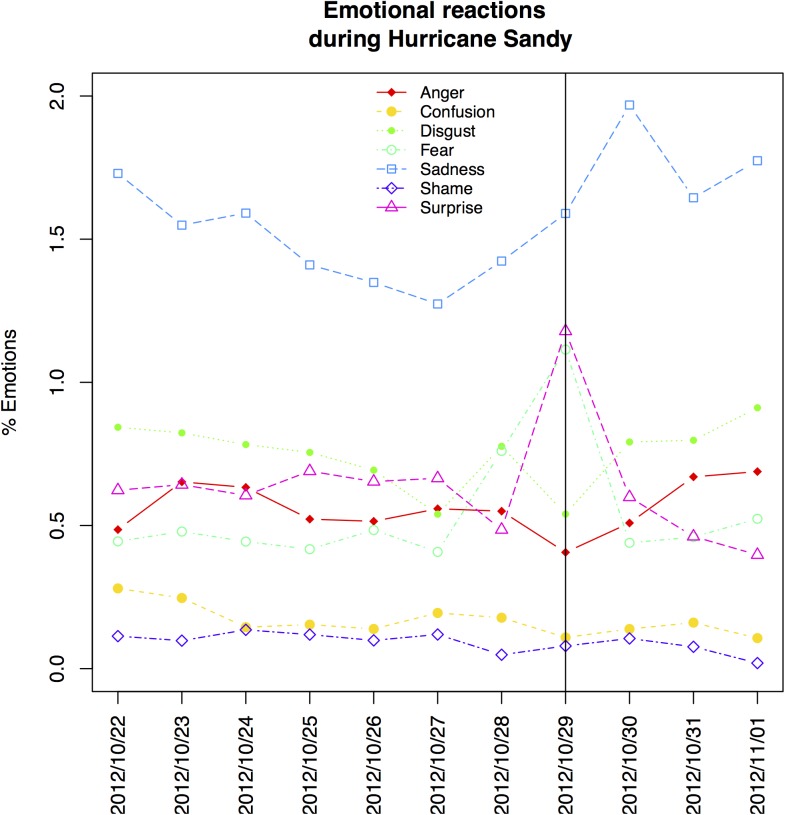
Percentages of emotions detected in Twitter tweets over the time of Hurricane Sandy between October 22 and November 1 2012. The storm hit the greater New York City area on October 29 2012.

**Fig 2:**
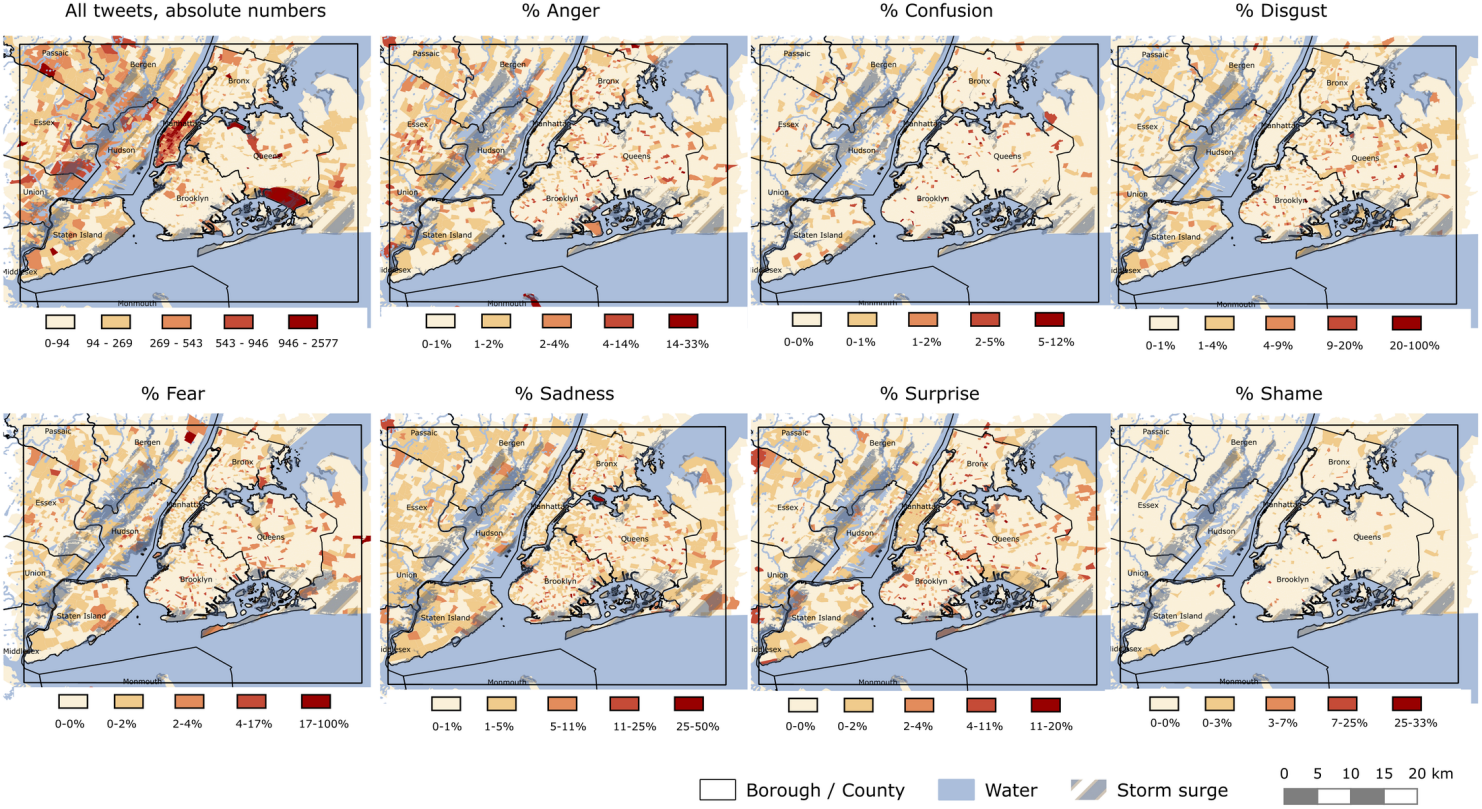
Spatial distributions of geo-located Twitter tweets and percentages of emotions classified in quintiles at the census tract level in the greater New York City area between October 22 and November 1 2012. Storm surge according to FEMA [59].

Specific emotions were extracted from the Twitter activity of users in the greater NYC area during Hurricane Sandy. We found 1,952 tweets (0.6%) classified as anger, 603 (0.2%) as confusion, 2,627 (0.8%) as disgust, 1,715 (0.5%) as fear, 5,457 (1.6%) as sadness, 350 (0.1%) as shame, and 2,192 (0.6%) as surprise. From Fig 1, we noted that sadness was the most pronounced emotion during the entire time frame and was particularly elevated one day after the hurricane made land fall in the greater NYC area. Percentages of fear and surprise were temporarily elevated in the Twitter activity of users on the day of disaster. We further noted that anger and disgust were slightly elevated in the three days after the disaster. Fig 2 describes the spatial distribution of absolute numbers of tweets and percentages of single emotions (displayed in quintiles) at the census tract level across the greater NYC area for the time frame of 11 days, i.e. from October 22 to November 1, 2012. We noted that most tweets were sent from locations that are highly populated (e.g., lower Manhattan), transit places (e.g., airports), or places of recreation (e.g., central park). Anger, fear, sadness, and surprise were elevated (highest quintile) in areas with a waterfront or in exposed areas (e.g., Sandy Hook, Englewood, Rikers Island, Cony Island) but to some extent also in scattered areas in the hinterland of New Jersey.

**Fig 3:**
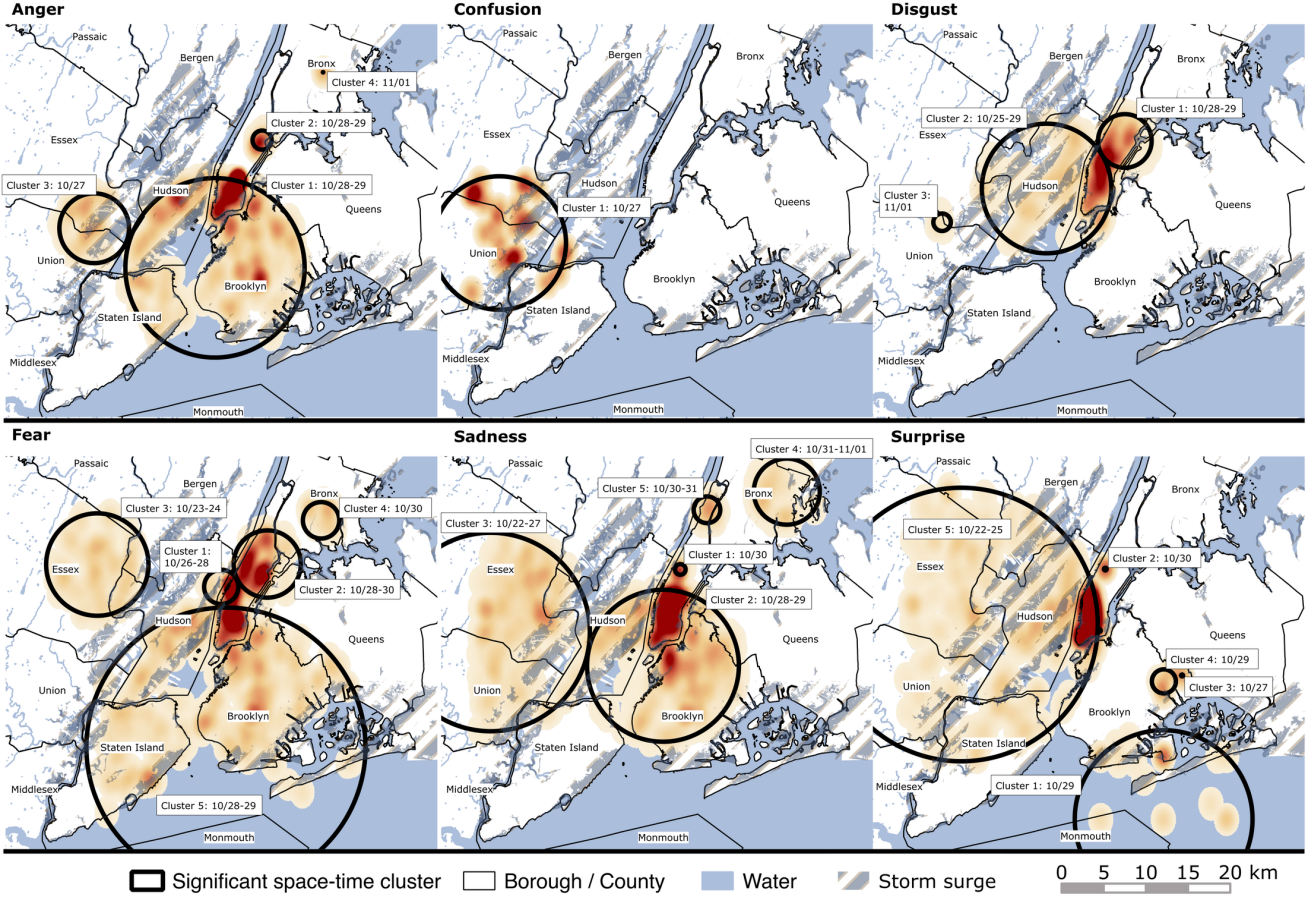
Scan statistic results for the time frame October 22 and November 1 2012. Circles indicate spatio-temporal clusters of excess risk of emotional reactions along with the time of emergence. Shaded areas within circles highlight geo locations of cluster-relevant tweets, with darker shading indicating higher densities. Storm surge according to FEMA [59].

Fig 3 and Table 1 provide information on excess risk of emotional reactions as indicated through space time clusters with information on the location, area of extent, date of emergence, duration, and significance of excess risk of basic emotions. In total, 25 significant space-time clusters were found: Four for anger, one for confusion, three for disgust, five for fear, five for sadness, and six for surprise. One post-disaster anger cluster was classified as a false positive, with users tweeting that they were at the “Mad Hatter Pub”. In the following, we primarily report on the most likely clusters in terms of statistical significance (cluster 1) representative of excess risk of basic emotions in space and time. Of these, anger, confusion, disgust, and fear appeared pre disaster in the days between October 26^th^–28^th^, surprise peri disaster on October 29^th^, and sadness emerged post disaster on October 30^th^. For anger, the most likely cluster had a radius of 11km and was found in areas across NYC’s downtown Manhattan, Brooklyn, North of Staten Island, and East of New Jersey’s (NJ) Hudson counties. This cluster included 63 cases over two days starting from October 28, that is, pre disaster, when about 24 were expected, resulting in a relative risk (RR) of 2.59. Examples of tweet texts within this cluster include “*[@…] this storm is ultimately blowing mines I'm mad [expletive] can't do nothing for 2 days but stay inna house*.” For confusion, we found one cluster with 7.9km radius including NJ’s adjacent counties Union, Essex, and Hudson, as well as Staten Island. The cluster included nine observed cases over one day starting October 27^th^ when about one case was expected (relative risk = 7.00). This cluster’s tweet texts included e.g., “*At #EWR*. *See you in 4 days #nyc*, *hello #ldn*. *Flying visit*. *Glad that [expletive] #Sandy left when she did*. *Chaotic scenes*.*”* For disgust, the most likely cluster had a radius of 3.2km across areas in uptown Manhattan and included 42 cases over two days starting October 28^th^ when 14 cases were expected (RR = 2.99). Within the disgust cluster, texts included “*The seaside boardwalk was destroyed this is horrible*.” Fear most likely clustered in Manhattan’s upper west side with a radius of 2km including 51 cases over three days starting October 26^th^ when about 15 cases were expected (RR = 3.31). Tweet texts included “*I'm really scared for this Hurricane*”. The most likely cluster for surprise had a radius of 10.5km, was found in areas of Coney Island and Rockaway Peninsula, and included 40 cases on one day (i.e. the day of the disaster on October 29^th^) when about five were expected (RR = 7.84). Texts included “*Still in disbelief that this is my front yard…”*. Sadness spatially clustered most significantly with a radius of 0.6km in Manhattan’s upper west side for one day on October 30^st^, that is, post disaster, when about eight cases were expected (RR = 6.91). Texts included e.g., “*Homes*, *devastated by fire and the effects of Hurricane Sandy*, *in the Breezy Point section of Queens NYC (Reuters)*.*”*

**Table 1:** Emotions classified by EMOTIVE (total N) and space-time scan statistic results. Statistical significant space-time clusters (i.e. spatio-temporal concentration of risk) of each emotion were found by the can statistic and were ordered according to the significance of the signal. Hence, cluster one of a particular emotion is the most likely cluster of that basic emotion and marked in bold. Note that *FP* refers to a false positive as identified from the tweet text.

Emotion (total N)	Cluster	Radius in km	Date of emergence	Number of days	Observed Cases	Expected Cases	Relative Risk	P value
**Anger (1,952)**	*FP*	*0*.*001*	*Oct 23*	*2*	*52*	*16*.*26*	*3*.*20*	*<0*.*001*
	**1**	**11**	**Oct 28**	**2**	**63**	**24.37**	**2.59**	**<0.001**
	2	1.1	Oct 28	2	14	1.61	8.73	<0.001
	3	4.1	Oct 27	1	14	2.33	6.02	<0.001
	4	0.02	Nov 1	1	6	0.26	23.57	<0.001
**Confusion (603)**	**1**	**7.9**	**Oct 27**	**1**	**9**	**1.29**	**7.00**	**<0.01**
**Disgust (2,627)**	**1**	**3.2**	**Oct 28**	**2**	**42**	**14.05**	**2.99**	**<0.001**
	2	7.6	Oct 25	5	233	165.05	1.41	<0.01
	3	1	Nov 1	1	6	0.29	20.96	<0.01
**Fear (1,715)**	**1**	**2**	**Oct 26**	**3**	**51**	**15.39**	**3.31**	**<0.001**
	2	3.9	Oct 28	3	84	44.36	1.89	<0.001
	3	6	Oct 23	2	52	23.41	2.22	<0.001
	4	2.1	Oct 30	1	13	2.21	5.87	<0.01
	5	16.5	Oct 28	2	138	89.45	1.54	<0.01
**Sadness (5,457)**	**1**	**0.6**	**Oct 30**	**1**	**53**	**7.67**	**6.91**	**<0.001**
	2	8.9	Oct 28	2	225	128.61	1.75	<0.001
	3	11.7	Oct 22	6	523	411.54	1.27	<0.001
	4	3.9	Oct 31	2	46	18.16	2.53	<0.001
	5	1.6	Oct 30	2	40	16.23	2.46	<0.05
**Surprise (2,192)**	**1**	**10.5**	**Oct 29**	**1**	**40**	**5.10**	**7.84**	**<0.001**
	2	0.2	Oct 30	1	17	1.54	11.02	<0.001
	3	0.1	Oct 27	1	9	0.48	18.61	<0.001
	4	1.4	Oct 29	1	15	2.28	6.58	<0.001
	5	16.1	Oct 22	4	381	290.60	1.31	<0.001
	6	0.2	Oct 29	1	8	0.87	9.21	<0.05

In addition, one cluster for fear (Table 1, fear cluster 2) persisted over three days from pre- to post-disaster (10/28-10/30). This cluster was located in uptown Manhattan, had a radius of 3.9km, and included 84 observed cases when about 44 were expected (RR = 1.89). Topics discussed in the tweet text included “*The storm is getting really harder am really scared I live next to a lot of trees am really scared they will fall*”, “*I'm officially a little nervous working next to this rattling window*!: *-O*”, or “*Power flicker*. *But still got to hear Wolf say*, *‘Live picture of a crane collapse that officials fear may collapse*.*’*”

Notably, many clusters included also tweet texts not directly related to the hurricane. Furthermore, some less significant clusters were comprised of messages from one user (anger cluster 4) or two users (disgust cluster 3) that were published from within the same area and within a relatively narrow time producing a signal. Since these clusters detected an emotion, we do not consider them as a false positive but nevertheless do not further interpret them.

## Discussion

We demonstrated a novel space-time syndromic surveillance approach for disaster mental health by extracting multiple emotions from Twitter for the greater NYC area over eleven days, October 22 to November 1, 2012, and detecting clusters that represented excess risk of these emotions in space and time as a way anticipating acute stress in the population.

We showed that EMOTIVE is able to detect multiple dimensions of emotional reactions in the population. This approach may have several utilities for disaster research. First, our study revealed for example that sadness and disgust were among the most commonly found negative emotions in the context of Hurricane Sandy. These basic negative emotions may inform about populations dealing with an immediate danger or experiencing an irrevocable loss, which is associated with human autonomous nervous system (ANS) responses [12]. Our detected emotional reactions may therefore inform about the distribution of distress in a population facing a natural disaster.

Second, we were able to show the distribution of these emotions before, during, and in the aftermath of disaster, and noted the times when the storm was considered most severe in the population of Twitter users. For example in our study, fear and surprise were elevated during the day Hurricane Sandy made landfall in the area (October 29, 2012), and sadness was particularly elevated on the day after. Our results are consistent with those of Kryvasheyeu et al., who showed that real and perceived threats, together with physical disaster effects, are directly observable through the intensity and composition of Twitter’s message stream [44].

Third, our approach allowed for the detection of excess risk of multiple basic emotions, along with the times, duration, locations, and spatial extent of these emotions. For example, we found a temporal dimension of excess risk in many basic emotions with most clusters identified for the time shortly before the hurricane made landfall. This may exhibit peoples’ emotional processing, dealing with the adaptation of (or coping with) a soon to arrive disaster expressed in anger, confusion, disgust, fear, and surprise, which mainly clustered before and during the storm. Tweet texts of Twitter users included discussions about service outfalls, possible crane collapses, the noises produced by the strong winds, high water levels, damage, and related tragedies, among others, with sometimes direct references made to the storm (e.g., using hash tags). In contrast, sadness most significantly clustered after the storm, which may indicate people’s processing of the adaptation to the consequences of the storm such as constraints (e.g., service shortage). Notably, the most significant sadness cluster (found in Manhattan for October 30^st^) included a tweet reflecting on a fire incident in Breezy Point, Queens, in which news outlets reported on over 100 housing units being destroyed by inundation and fire [5,56].

Not only did we identify basic emotions during these times, we additionally identified excess risk that may further indicate intense processing of these emotions over the course of only a few days around the day on which the hurricane made landfall in the area, which may be related to acute stress. However, our study was conducted at the tweet level rather than the individual Twitter user level and further studies are needed to provide evidence whether excess risk of negative emotions (e.g., fear) over the course of several days is comparable to experiencing acute stress in this context.

We further found a spatial dimension of excess risk of basic negative emotions, that is, that intense emotional reactions were not equally distributed across the study area, but rather were locally dependent. Our study results are noteworthy as they exhibit, for the first time, space-time clusters (of excess risk of basic emotions) in which environmental threats are discussed, which were locally and temporally related to the storm (e.g., strong winds, falling trees, high water). This information may be useful to identify the role of neighborhood socio-ecological factors in shaping risk, risk that is potentially shared by a larger community including also populations who are not Twitter users but who live in the same area in which respective tweets caused a signal. As such, our approach also provides useful information on how the disaster event was perceived, either directly or indirectly and could help inform public health interventions to ensure that vulnerable neighborhoods are well prepared for traumatic events and build resilient populations. Our approach may also be informative about the risk for long-term consequences of disasters. For example, early emotional reactions may predict longer-term mental health needs [4], so that this approach could also assist in the detection of mental health needs over time and allocation of services in the long-term aftermath of a disaster and further studies are needed to test and evaluate this approach. In addition, a prospectively applied space-time surveillance approach based on social media data could detect excess risk of emotional reactions including real-time information on risk, location, spatial extent, time of occurrence, and duration of basic emotions in populations facing disaster. Future work may create risk maps based on this information to identify neighborhoods and communities for the immediate intervention and provision of mental health and other services of need in these areas. In countries or areas without efficient emergency infrastructure, a space-time syndromic surveillance approach as outlined here may have potential for the early detection of mental health risk during disasters and to guide emergency care and rescue efforts in the aftermath of these events. Research is therefore needed to achieve the full potential of this approach in these countries or areas.

All the above suggests that a space-time surveillance approach for mental health based on social media data can support traditional surveillance approaches providing useful additional information in relation to the event under study. However, more studies are needed to better control for bias, identify associations with reliable and valid instruments measuring mental health, and to explore computational methods for continued model-fitting, causal relationships, and ongoing evaluation of big data analysis in this and other contexts [37,38]. Further work may therefore address the limitations to our study. First, we only included tweets posted in English because the program used for sentiment analysis could only accommodate English text. Since the majority of the tweets in this area over the study period were in English, we do not anticipate strong bias by excluding other languages. However, subsequent analyses would benefit from translating additional languages into English or developing programs that can handle multiple languages. Second, we only included textual Twitter data and future research could incorporate data from other social media outlets (e.g., Facebook, YouTube, or Sina Weibo) and develop algorithms to code for the emotional content of visual images and videos. Third, only tweets with information on their geographic locations were used and thus are not representative of all Twitter users in the investigated area. This could explain in part why the majority of the tweets in our analysis were in English, since demographic variations exist in those who geotag their tweets [57]. Lack of representativeness, however, is inherent to geospatial analysis using social media data, and it is notable that our study detected clusters of emotional responses despite this limitation. Further studies could also include information about the place to which the tweet is referring (e.g., the place ID tag in Twitter or from the tweet text) that could be used in detecting global clustering in the data before looking at the specific location and quality of the cluster itself. Fourth, it should be kept in mind when interpreting our findings that users might have moved from one place to another while texting, or may have been talking about areas other than the ones in which they were living, which may introduce possible spatial or temporal lags in the signal, as was shown with the sadness cluster in Manhattan that contained one tweet reflecting on the fire incident in Breezy Point, Queens. Fifth, it is worth mentioning that the distribution of some emotions may also be a proxy for other emotions in space and time and further studies should control for this with multivariate scan statistics [58], for example. Lastly, due to limited computation times, our temporal resolution was a one-day time frame and results could have been different had we conducted a more fine-grained (e.g., minute-by-minute) temporal analysis.

Notwithstanding these limitations, the results demonstrate the potential for a novel space-time syndromic surveillance approach for mental health that is able to detect multiple dimensions of negative emotional reactions in the context of disaster, temporal changes in emotional reactions, and geographic concentrations of excess risk of negative basic emotions pre, peri, and post disaster as a way to anticipate acute stress in the population. Maps can thereby be generated to identify the areas most at risk of population mental health problems after mass traumatic events so that we know where we may most effectively intervene to help reduce the mental health consequences of disasters. We expect our study to be a starting point for the evolution of more fully elaborated models that can prospectively detect mental health risk during and after disasters using real-time social media data. In countries without efficient emergency infrastructure, this approach may also have potential for the early detection of the mental health consequences of mass traumatic events and to guide emergency care and rescue efforts. As such, social media data present a real opportunity for mental health surveillance after large-scale disasters to help identify areas of mental health needs.
